# Life-Threatening Hypokalemic Paralysis in a Young Bodybuilder

**DOI:** 10.1155/2014/483835

**Published:** 2014-02-11

**Authors:** Kitty K. T. Cheung, Wing-Yee So, Alice P. S. Kong, Ronald C. W. Ma, Francis C. C. Chow

**Affiliations:** Departments of Medicine and Therapeutics, Prince of Wales Hospital, The Chinese University of Hong Kong, 30-32 Ngan Shing Street, Shatin, New Territories, Hong Kong

## Abstract

We report a case of life-threatening hypokalemia in a 28-year-old bodybuilder who presented with sudden onset bilateral lower limbs paralysis few days after his bodybuilding competition. His electrocardiogram (ECG) showed typical u-waves due to severe hypokalemia (serum potassium 1.6 mmol/L, reference range (RR) 3.5–5.0 mmol/L). He was admitted to the intensive care unit (ICU) and was treated with potassium replacement. The patient later admitted that he had exposed himself to weight loss agents of unknown nature, purchased online, and large carbohydrate loads in preparation for the competition. He made a full recovery after a few days and discharged himself from the hospital against medical advice. The severe hypokalemia was thought to be caused by several mechanisms to be discussed in this report. With the ever rising number of new fitness centers recently, the ease of online purchasing of almost any drug, and the increasing numbers of youngsters getting into the bodybuilding arena, clinicians should be able to recognize the possible causes of sudden severe hypokalemia in these patients in order to revert the pathophysiology.

## 1. Introduction

Severe hypokalemia can be life threatening. It can be caused by genuine diseases or iatrogenic. Either way, prompt recognition of the condition and the underlying causes is needed for effective management of the patients. With the ever rising number of new fitness centers recently, the ease of online purchasing of almost any drug, and the increasing numbers of youngsters getting into the bodybuilding arena, clinicians should be able to recognize the possible iatrogenic causes of sudden severe hypokalemia in this group of patients.

## 2. Case Presentation

**Figure 1:**
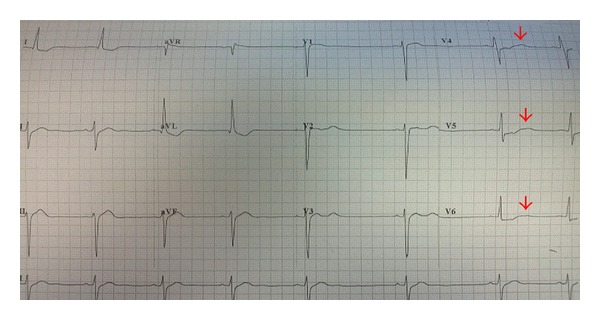
ECG of the patient showing typical u-waves of hypokalemia.

A 28-year-old Chinese man with good past health and no significant family history presented to the Accident and Emergency Department (AED) due to sudden onset bilateral lower limb paralysis (Medical Research Council power grading 2/5) on August 13, 2013. The patient reported no recent head, neck, or spine injury and remained conscious all along. Physical exam revealed a muscular young man with no features of dehydration, hyperthyroidism, or Cushing's syndrome. Urgent imaging including plain computer tomography (CT) of brain and X-ray spine showed no abnormality. ECG showed typical u-waves and serum biochemistries came back to show severe hypokalemia (1.6 mmol/L, RR 3.5–5.0 mmol/L) (Figure 1). His serum creatinine, magnesium, thyroid stimulating hormone, and pH levels were all normal. He was therefore admitted to the ICU and was commenced on oral and intravenous potassium replacement. Spot urine for potassium (14 mmol/L) was only saved after the start of potassium replacement when the paired serum potassium was 2.2 mmol/L. In total, 20 mmol of intravenous and 10 mmol of oral potassium chloride were given, and his serum potassium was stabilized at 3.7–4 mmol/L eighteen hours after admission. Urine for toxicology screen was saved and the report was pending at that time.

Upon further questioning, the patient admitted to have taken some weight loss agents of unknown nature which he had purchased over the Internet until the date of his competition. He then disclosed that, in preparation for the bodybuilding competition, he had been taking heavy loads of carbohydrate for muscle building and restricting salt and fluid intake a few days prior to the presentation. He denied any recent use of anabolic steroid. The patient recovered fully from his paralysis a couple of days later and discharged himself from the hospital against medical advice on August 15, 2013. His latest serum potassium prior to discharge was 4.4 mmol/L.

The urine toxicology screen came back two weeks after the patient's presentation showing rhein, emodin, aloe-emodin, and physcion. Unexpectedly, mycophenolate mofetil (MMF) and diltiazem were also identified although the patient denied having any prescribed medications. The patient was phoned up and the urine toxicology results were explained to him, with emphasis on the potential adverse effects of taking drugs without prescription from doctors.

## 3. Discussion

It is well known that in preparation for bodybuilding competitions, bodybuilders, in addition to hard muscle training, very often engage themselves in dietetic manipulations. Three phases have been described. The first phase involves few months of hypercaloric nutrition rich in proteins, for the build-up of muscle mass. The second phase is a period of reduced caloric intake to reduce subcutaneous fat. The third phase, during the last week of preparations, includes simultaneous extreme carbohydrate intake to load muscles with glycogen, sodium, and water restriction to produce subcutaneous volume deficit and better definition of muscle contours. Hypokalemia and flaccid paralysis often result during the course of these dietetic manipulations [1].

Our patient appeared to have been following these phases in preparation for his bodybuilding competition and suffered from the resulting hypokalemic paralysis. The intake of extreme carbohydrate loads had led to an intracellular shift of the potassium from extracellular to intracellular space through stimulating endogenous insulin release, which in turn promoted the entry of potassium into skeletal muscle and liver cells by activating the Na-K-ATPase pump [2]. The quick reversal of his potassium level with just 20 mmol of intravenous and 10 mmol of oral potassium chloride made this a possible explanation as it is classical for hypokalemia due to intracellular shift to be reverted quickly without large amount of potassium replacement.

The use of “weight loss agents” might have included diuretics, such as spironolactone or other mineralocorticoid antagonists, which competed with aldosterone for receptor sites in the distal renal tubules, increasing in sodium chloride and water excretion while conserving potassium and hydrogen ions. The sudden withdrawal of spironolactone right after the competition might have caused a relative mineralocorticoid excess, since mineralocorticoid was previously overstimulated due to its action being blocked in the renal tubules. This relative mineralocorticoid excess therefore contributed to the hypokalemia. Since the half-life of spironolactone in serum is only 78–84 minutes and is only found in the body for two-three days after ingestion, this might be the reason for it not being detected in the urine toxicology screen [3].

Rhein, emodin, aloe-emodin, and physcion are anthraquinones with laxative effect, and using these chronically may cause hypokalemia [4]. Therefore, these, most likely constituents of the “weight loss agents” from over the Internet, serve as the third possible cause of his severe hypokalemia. Although MMF and diltiazem could not be accounted for the life-threatening hypokalemia, they serve to remind all clinicians that everything could be present in over the Internet drugs, including those which should not be supplied without doctor's prescription.

In conclusion, we report here a young bodybuilder who presented with life-threatening hypokalemic paralysis few days after his bodybuilding competition. Three possible mechanisms for the severe hypokalemia were identified: (1) intracellular potassium shift due to extreme carbohydrate loads (2) suspected spironolactone, or other mineralocorticoid antagonists, content in the “weight loss agents” purchased by patient over the Internet leading to relative mineralocorticoid excess shortly after withdrawal (3) anthraquinones with laxative effect leading to gastrointestinal loss of potassium. With the ever rising number of new fitness centers recently, the ease of online purchasing of almost any drug, and the increasing numbers of youngsters getting into the bodybuilding arena, clinicians should be able to recognize the possible causes of sudden severe hypokalemia in these patients in order to treat and advise the patients efficiently.
